# The Influence of Obesity on Nutrition and Physical Activity during COVID-19 Pandemic: A Case-Control Study

**DOI:** 10.3390/nu14112236

**Published:** 2022-05-27

**Authors:** Mariusz Wyleżoł, Beata I. Sińska, Alicja Kucharska, Mariusz Panczyk, Filip Raciborski, Dorota Szostak-Węgierek, Magdalena Milewska, Bolesław Samoliński, Mariusz Frączek, Iwona Traczyk

**Affiliations:** 1 Department of General, Vascular and Oncological Surgery, Medical University of Warsaw, 61 Żwirki i Wigury Street, 02-091 Warsaw, Poland; mariusz.wylezol@wum.edu.pl (M.W.); mariusz.fraczek@wum.edu.pl (M.F.); 2 Warsaw Obesity Center, Czerniakowski Hospital, 19/25 Stępińska Street, 00-739 Warsaw, Poland; 3 Department of Human Nutrition, Faculty of Health Sciences, Medical University of Warsaw, 27 Erazma Ciołka Street, 01-445 Warsaw, Poland; beata.sinska@wum.edu.pl (B.I.S.); iwona.traczyk@wum.edu.pl (I.T.); 4 Department of Education and Research in Health Sciences, Faculty of Health Sciences, Medical University of Warsaw, 14/16 Litewska Street, 00-581 Warsaw, Poland; mariusz.panczyk@wum.edu.pl; 5 Department of Prevention of Environmental Hazards and Allergology, Faculty of Health Sciences, Medical University of Warsaw, 1a Banacha Street, 02-091 Warsaw, Poland; filip.raciborski@wum.edu.pl (F.R.); boleslaw.samolinski@wum.edu.pl (B.S.); 6 Department of Clinical Dietetics, Faculty of Health Sciences, Medical University of Warsaw, 27 Erazma Ciołka Street, 01-445 Warsaw, Poland; dorota.szostak-wegierek@wum.edu.pl (D.S.-W.); mmilewska@wum.edu.pl (M.M.)

**Keywords:** COVID-19, obesity, nutrition, pandemic

## Abstract

Obesity is one of the important risk factors for a severe course of COVID-19. Maintaining a healthy body weight through diet and physical activity is a reasonable approach to preventing a SARS-CoV-2 infection or in alleviating its course. The goal of the study was to determine the influence of obesity on nutrition and physical activity during the COVID-19 pandemic. A total of 964 respondents, including 227 individuals with a body mass index (BMI) ≥30 kg/m^2^ were evaluated in this study. In the case of 482 respondents, including 105 individuals (21.8%) with BMI ≥ 30 kg/m^2^, the data were collected during the pandemic period from 1 June to 31 August 2020. The remaining 482 individuals were the “pre-pandemic” group, selected via propensity score matching (PSM) out of the 723 National Health Program study participants whose data was collected in 2017–2019. The evaluated dietary health factors were quantitatively similar in patients with BMI of either <30 kg/m^2^ or ≥30 kg/m^2^ and showed no significant changes during the pandemic. The diets of those who suffered from obesity prior to the pandemic showed the evaluated unhealthy nutritional factors to be less pronounced in comparison with those of individuals with BMI < 30 kg/m^2^. During the pandemic, the BMI ≥ 30 kg/m^2^ group showed a significant increase in the overall calorie intake (by 319 kcal; *p* = 0.001) and an increased consumption of total carbohydrates 299.3 ± 83.8 vs. 252.0 ± 101.5; *p* = 0.000), sucrose (51.7 ± 30.0 vs. 71.6 ± 49.9; *p* = 0.000), plant protein (26.3 ± 12.1 vs. 29.3 ± 8.3; *p* = 0.040), total fat (73.1 ± 42.6 vs. 84.9 ± 29.6; *p* = 0.011) and saturated fatty acids (29.5 ± 16.4 vs. 34.3 ± 13.9; *p* = 0.014) in comparison with the pre-pandemic period. The energy and nutritional value of the diets of BMI < 30 kg/m^2^ individuals did not change between the pre-pandemic and pandemic period. Before the pandemic, the level of leisure physical activity of the BMI ≥ 30 kg/m^2^ group was significantly lower than of those with BMI < 30 kg/m^2^. Such differences were not observed in the levels of physical activity at work or school. The pandemic did not alter the amount of physical activity either during leisure time or at work/school in individuals with BMI ≥ 30 kg/m^2^. However, respondents without obesity exercised significantly less during the pandemic than before. In conclusion, the pandemic altered the diets and levels of physical activity in the Polish population, with dietary changes observed in individuals with BMI ≥ 30 kg/m^2^ and changes in physical activity observed in those with BMI < 30 kg/m^2^.

## 1. Introduction

The mutual, unfavorable relationships between obesity and the COVID-19 pandemic are well known. Obesity has been linked to a significant increase in morbidity and mortality from COVID-19 [1,2,3]. At the same time, the pandemic has exacerbated the problem of obesity, as evidenced by the commonly observed weight gain [4,5].

However, if we begin to analyze in detail the causes of the negative impact of the pandemic on obesity, the answer to why this is happening is not so obvious due to conflicting study results, including the impact of the pandemic on weight gain.

For example, Chinese authors found that individuals who did not suffer from overweight or obesity had less awareness of weight gain under semi-lockdown conditions [6]. Conversely, a study conducted in Lithuania demonstrated that people with higher BMI values gained weight more often compared with those with normal BMI values [7].

Interestingly, it was demonstrated that people with obesity had the largest increase in healthy eating behaviors compared to normal-weight and overweight individuals under stay-at-home orders [8].

Based on a cross-sectional online survey performed in Poland, almost 30% of the studied population experienced weight gain, with 43% and nearly 52% of respondents reporting eating and snacking more, respectively [9]. Another Polish study also demonstrated a negative influence of the COVID-19 pandemic on body weight [10]. The authors found that the percentage of people snacking between meals increased and that eggs, potatoes, sweets, canned meat and alcohol were consumed considerably more often during lockdown, while fast-food products, instant soups and energy drinks were consumed significantly less frequently. There was a marked decrease in the number of daily servings of the following products: bakery products, red meat, fast foods, instant soups, sweet beverages and energy drinks. Conversely, the number of daily servings of sweets and canned meat significantly increased. According to a study performed by scientists from the United Kingdom, individuals with obesity were most likely to report declines in weight gain protective behaviors [11].

Importantly, effective treatment of obesity has been associated with a reduced risk of death and invasive mechanical ventilation related to COVID-19 [12,13]. However, it was demonstrated that stay-at-home orders led to more difficultly in achieving weight loss goals, reduced exercise time and intensity, and increased stockpiling of food and stress eating among people with obesity [14].

To the best of our knowledge, all of these studies were based on the respondents recalling the details on their nutrition and physical activity from before the COVID-19 pandemic. Therefore, all of those studies are likely to be burdened with recall bias, and we know that the longer the time period is, the more likely patients are to “telescope” forward or backward or to mistakenly believe the recalled event occurred more or less recently than it did.

We realize that thanks to a Polish national survey, which was planned to be conducted in 2017–2020, we had a unique opportunity to compare actual data from before the pandemic and from a period during the COVID-19 pandemic, and therefore, we believe that the achieved results can be more representative and reliable in comparison with the often contradictory results of other studies, as presented above.

The goal of our study was to determine the influence of obesity on nutrition and physical activity during the COVID-19 pandemic. These results can inform clinicians and healthcare professionals about effective strategies to prevent weight gain in patients with obesity, especially during the current or potential future pandemics.

## 2. Materials and Methods

### 2.1. Design

The data analyzed in our study came from two representative cross-sectional studies of the dietary habits and nutritional status of the adult Polish population conducted over the period 2017–2020 [15].

### 2.2. Participants

The analyses conducted as part of this study were conducted on the data obtained from respondents during the summer months (1 June–31 August 2020) of the COVID-19 pandemic (the Pandemic group) and in a pre-pandemic period (the PrePandemic group). Given the incomplete balance of the groups, the main analysis included 482 participants who were matched using propensity score matching (PSM), based on such demographic variables as the age, sex, education, place of residence, BMI and the number of people in the household. Individuals from the PrePandemic group and those from the Pandemic group were matched with the nearest neighbor matching method. Our analysis included respondents aged 19–75 years. The upper age limit was adopted due to the possible advanced-age-related limitations in easy communication and fact recollection due to increased rates of neurodegenerative conditions.

### 2.3. Data Collection

The study was conducted by experienced interviewers who had been trained by the study team. The same methodology was used in both evaluated periods (pre-pandemic and pandemic), the only difference being in the way of data collection. Prior to the pandemic, the questionnaires were completed directly in respondents’ homes via computer-assisted personal interviews (CAPI), whereas during the pandemic, in-person interviews were replaced by telephonic ones (computer-assisted telephone interviews, CATI). The measurement of anthropometric parameters in person was replaced by anthropometric data being reported by respondents.

In order to assess the effects of obesity on the eating habits and physical activity of the Pandemic group, both study groups (PrePandemic and Pandemic) were stratified by BMI values (BMI < 30 kg/m^2^ and BMI ≥ 30 kg/m^2^) [16]. This division yielded four study subgroups: PrePandemic BMI < 30 kg/m^2^, PrePandemic BMI ≥ 30 kg/m^2^, Pandemic BMI < 30 kg/m^2^ and Pandemic BMI ≥ 30 kg/m^2^.

In order to exclude the potential effect of concomitant diseases on eating habits and physical activity, a comparative analysis of the rates of cardiovascular, respiratory, gastrointestinal, endocrine and musculoskeletal conditions, as well as malignancies, diabetes and allergy was performed. Information on the occurrence of diseases was collected based on respondents’ declarations. Respondents selected the diseases they had experienced from a given list.

### 2.4. Instruments

The following tools, instruments, scales and measurements were used in this study.

#### 2.4.1. Weight and Height Measurements

Prior to the pandemic, the weight and height of respondents were measured according to the established standards [17,18]. During the pandemic, respondents declared their current body weight and height values. The resulting data helped calculate the BMI (kg/m^2^) values.

#### 2.4.2. Eating Habit Assessments

The methodology adopted for our study was based on the European Food Safety Authority guidelines on the EU Menu [19,20] and on the Polish Academy of Sciences Committee on Human Nutrition Science recommendations [21,22].

Two methods of assessing eating habits were used. The fundamental assessment tool was an interview regarding the food consumed over the previous 24 h (24 h dietary recall) conducted with each respondent twice, at least 5 days apart. In order to allow for the possible eating habit variability throughout the week, assessments were conducted on different days of the week. The portion sizes and types of dishes were declared by the respondents based on their at-home measurements and the information printed on product labels. During the pre-pandemic period, they were additionally based on photographs of portion sizes and dishes, which—along with the national database on the nutritional value of foods and national nutritional norms—are an integral part of the DIETA 6.0 software [23], which can be used for calculating the nutritional value of diets.

A food frequency questionnaire (FFQ) was additionally used in order to identify intra-individual variability in food consumption (e.g., during holidays or vacations) and to minimize its confounding effects on the analyzed data. For the purpose of this study, a simplified version of the FFQ was used, one composed based on a validated KomPAN questionnaire [24]. There were 20 items addressing the frequency of food consumption. The consumption frequency of particular foods was expressed with the following weighted values: 0.00 (never), 0.06 (1–3 times a week), 0.14 (once a week), 0.50 (several times a week), 1.00 (daily), 2.00 (several times a day).

In order to assess the quality of nutrition, we used the pro-healthy diet indicator (PHDI) and non-healthy diet indicator (NHDI) developed for the purposes of this study. The PHDI was based on 8 healthy products/food types, and the NHDI was based on 12 products/food types whose consumption should be limited (Table 1). The consumption of each product/food type with the frequency specified in Table 1 yielded 1 point, with the maximum score of 8 points (the higher the score, the more closely the diet reflected the recommended standards). Analogically, the maximum score for a non-healthy diet was 12 points (the higher the score, the further the diet deviated from the recommended nutritional standards) [25].

#### 2.4.3. Physical Activity Assessment

The level of physical activity was assessed based on the KomPAN questionnaire developed by the Behavioral Conditions of Nutrition Team of the Polish Academy of Science Committee of Human Nutrition Science [26]. The assessment of physical activity based on this questionnaire is subjective and includes two domains. One domain involves the levels of physical activity at work or school, and the other domain—the physical activity levels at home. Both domains are stratified into three levels of physical activity shown in Table 2.

### 2.5. Ethical Considerations

This study was approved by the Ethical Review Board at the Medical University of Warsaw (approval No. AKBE/163/17 and AKBE/164/17). The study was conducted in accordance with the General Data Protection Regulation.

### 2.6. Data Analysis

The study population was characterized with descriptive statistics. Quantitative variables were expressed as measures of central tendency (mean (M) and median (Mdn)), dispersion (standard deviation (SD), interquartile range (IQR)) and position (upper and lower quartile (Q1 and Q3)). Categorical variables were expressed as numbers (*n*) and proportions (%).

If the type of the dependent variable allowed, null hypotheses were tested with parametric statistics. As a general rule, sample sizes equal to or greater than 30 are deemed sufficient for the central limit theorem to hold, meaning that the distribution of the sample means is fairly normally distributed [27]. Depending on the type of the dependent variable, the PrePandemic and Pandemic groups were compared with the use of the chi-squared test (χ^2^ test), Student’s *t*-test or analysis of variance (ANOVA) with Fisher’s least significant difference test (LSD test). The effect size was determined with either Cohen’s *d* or eta-squared, with a 95% confidence interval.

All statistical calculations were performed using STATISTICA software version 13.3 (TIBCO Software, Palo Alto, CA, USA). Two-sided *p* < 0.05 was considered statistically significant for all null hypotheses tested.

## 3. Results

### 3.1. Participant Characteristics

**Figure 1:**
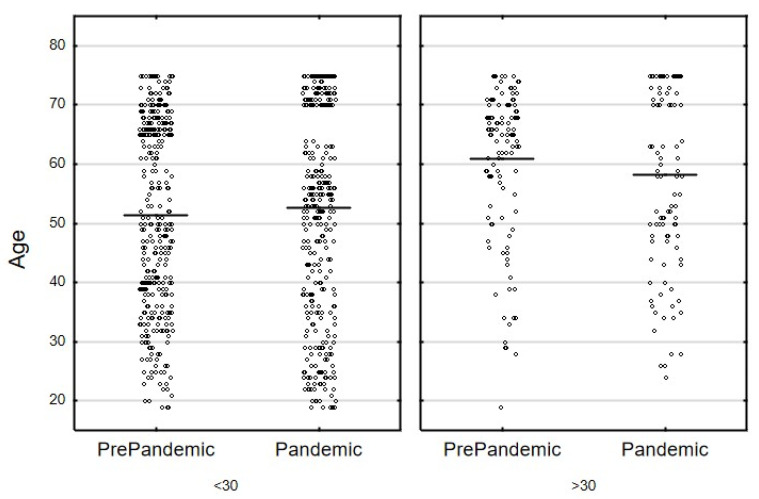
Distribution of the age variable in the PrePandemic vs. Pandemic samples stratified by the BMI category (<30 vs. ≥30) (two-way ANOVA: F_(1, 960)_ = 2.538; *p* = 0.111, horizontal lines illustrate the means).

**Figure 2:**
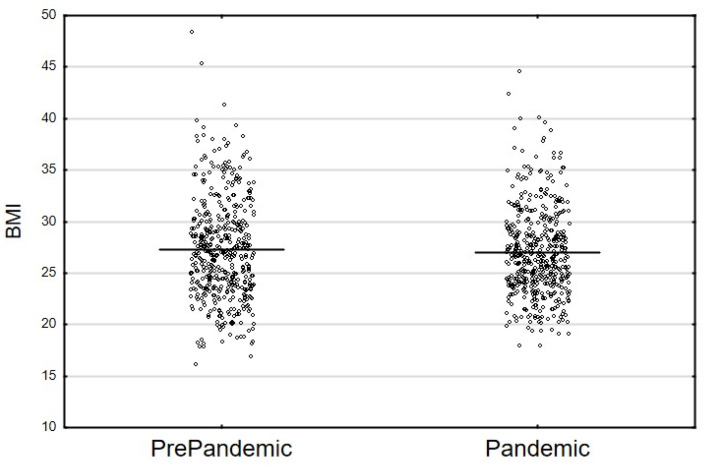
Distribution of the BMI variable in the PrePandemic vs. Pandemic samples (Student’s *t*-test: *t*_(962)_ = 1.059; *p* = 0.290, horizontal lines illustrate the means).

A total of 964 participants were evaluated, including 227 individuals with BMI ≥ 30 kg/m^2^. The data from 482 participants, including 105 individuals (21.8%) with BMI ≥ 30 kg/m^2^, were collected during the COVID-19 pandemic (1 June–31 August 2020). The data from the pre-pandemic period were collected from another 482 individuals (who were matched with the use of PSM). The latter group was part of the total of 723 National Health Program study participants whose data had been collected in the period 2017–2019. Study group characteristics were presented in Table 3. The study groups showed no differences in terms of sex, education, marital status, household finances, the number of people per household (Table 3), age (Figure 1) or BMI (Figure 2).

The rates of cardiovascular, respiratory, gastrointestinal, endocrine, musculoskeletal disorders and malignancies, diabetes and allergies in BMI ≥ 30 kg/m^2^ individuals were comparable before and during the pandemic. However, gastrointestinal and respiratory disorders were found to be more common in Pandemic BMI < 30 kg/m^2^ individuals (Table 4).

### 3.2. Physical Activity

**Figure 3:**
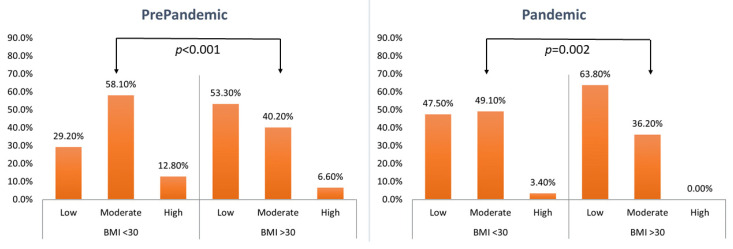
Assessment of physical activity during leisure time (Mann–Whitney–Wilcoxon test).

**Figure 4:**
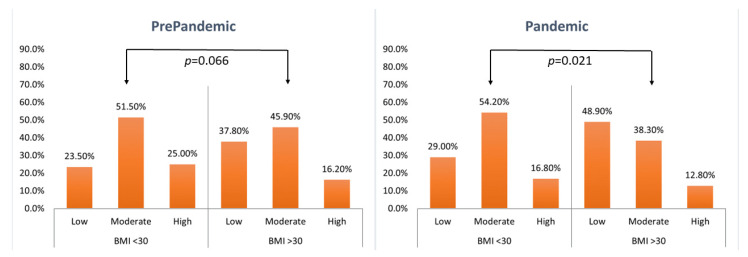
Assessment of physical activity at work or school (Mann–Whitney–Wilcoxon test).

The data on physical activity during leisure time were presented in Table 5 and Figure 3 and the data on physical activity at work/school—in Table 6 and Figure 4. The declared level of leisure physical activity in individuals with BMI ≥ 30 kg/m^2^ prior to the pandemic was significantly lower than that reported by individuals with BMI < 30 kg/m^2^. Such differences were not observed for physical activity at work/school. The BMI ≥ 30 kg/m^2^ subgroup showed no effect of the pandemic on the level of physical activity either during leisure time or at work or school. Conversely, individuals with BMI < 30 kg/m^2^ declared reduced levels of physical activity during the pandemic both during leisure time and at work or school. Despite the observed reduction in the levels of physical activity in BMI < 30 kg/m^2^ individuals during the pandemic, their physical activity (both during leisure time and at work/school) was still higher than that in BMI ≥ 30 kg/m^2^ individuals.

### 3.3. Nutritional Changes

#### 3.3.1. Qualitative Changes

PHDI analysis revealed comparable scores in the BMI < 30 kg/m^2^ and BMI ≥ 30 kg/m^2^ subgroups both prior to and during the pandemic. Moreover, the BMI-based subgroups showed no significant changes in PHDI during the pandemic (Table 7).

NHDI analysis showed the scores in the PrePandemic BMI ≥ 30 kg/m^2^ subgroup to be significantly lower than those in the PrePandemic BMI < 30 kg/m^2^ subgroup (4.06 ± 1.48 vs. 4.84 ± 2.13, LSD test: *p* = 0.003), which meant fewer unhealthy factors in the diet of respondents with obesity.

The pre-pandemic and pandemic eating habits showed no changes in terms of NHDI in individuals with BMI < 30 kg/m^2^. However, there was a significantly lower consumption frequency of refined grains, lard, read and processed meat during the pandemic (Table 6). The mean NHDI scores in the Pandemic BMI ≥ 30 kg/m^2^ subgroup increased significantly in comparison with those in the corresponding PrePandemic subgroup (4.58 vs. 4.06, respectively; LSD test: *p* = 0.049) and reached the values similar to those in the Pandemic BMI < 30 kg/m^2^ subgroup (4.58 vs. 4.67, respectively; LSD test: *p* = 0.685; Table 5). This detrimental change in NHDI scores in respondents with BMI ≥ 30 kg/m^2^ may have been due to an increased consumption of fried foods (Table 8).

#### 3.3.2. Quantitative Changes

During the pre-pandemic period, out of the two BMI-based subgroups, a higher calorie intake was observed in individuals with BMI < 30 kg/m^2^, with the daily difference in calorie intake of 223 kcal (LSD test: *p* = 0.005). The BMI ≥ 30 kg/m^2^ subgroup showed a significantly higher calorie intake of 319 kcal (LSD test: *p* = 0.001) during the pandemic, which became comparable to the calorie intake in the Pandemic BMI < 30 kg/m^2^ subgroup (2290 ± 591 vs. 2217 ± 695, respectively; LSD test: *p* = 0.370).

The Pandemic BMI ≥ 30 kg/m^2^ subgroup showed an increase in total carbohydrate consumption in comparison with the corresponding PrePandemic subgroup (299.3 ± 83.8 vs. 252.0 ± 101.5, respectively; LSD test: *p* = 0.000).

Sucrose consumption by individuals with BMI ≥ 30 kg/m^2^ also showed changes. Prior to the pandemic, sucrose consumption in BMI ≥ 30 kg/m^2^ individuals was significantly lower than in BMI < 30 kg/m^2^ individuals (51.7 ± 30.0 vs. 58.3 ± 33.0, respectively; LSD test: *p* = 0.002). During the pandemic, sucrose consumption was higher in BMI ≥ 30 kg/m^2^ individuals (PrePandemic BMI ≥ 30: 51.7 ± 30.0 vs. Pandemic BMI ≥ 30: 71.6 ± 49.9, LSD test: *p* = 0.000). We would like to emphasize that, conversely to the pre-pandemic period, when sucrose consumption was lower in individuals with BMI ≥ 30 kg/m^2^ than in those with BMI < 30 kg/m^2^, during the pandemic, the situation was reversed, with higher sucrose consumption reported by BMI ≥ 30 kg/m^2^ individuals than by those with BMI < 30 kg/m^2^ (62.6 ± 39.5 vs. 71.6 ± 49.9, respectively; LSD test: *p* = 0.03).

Analysis of the pre-pandemic and pandemic period also revealed differences in respondents with obesity in terms of the consumption of plant protein (26.3 ± 12.1 vs. 29.3 ± 8.3, respectively; LSD test: *p* = 0.040), total fat (73.1 ± 42.6 vs. 84.9 ± 29.6, LSD test: *p* = 0.011) and saturated fatty acids (29.5 ± 16.4 vs. 34.3 ± 13.9, LSD test: *p* = 0.014). However, the pandemic seemed to have no effect on total protein, animal protein or dietary fiber consumption (Table 9).

The calorie and nutritional intake in the diet of Pandemic BMI < 30 kg/m^2^ individuals were not different than those in the corresponding PrePandemic group.

## 4. Discussion

Our study showed that the pandemic brought changes in the dietary habits and levels of physical activity of the Polish population. Interestingly, these changes were not the same in the whole study population but rather were obesity related. Individuals with BMI ≥ 30 kg/m^2^ reported changes in their diets, whereas individuals with BMI < 30 reported lower levels of physical activity.

Our study demonstrated that the progressive weight gain in people with obesity observed in other studies was due to increased food consumption and unfavorable nutritional choices. The increase in food intake was demonstrated by the increased total fat and carbohydrate consumption and markedly increased calorie intake that were typical of the diets during the pandemic. We also observed a significant increase in the consumption of sucrose, despite the unchanged consumption of sweets and sweetened drinks. The increase in sucrose intake may be associated with the increased consumption of fried foods. Possibly, people with obesity introduced more fried, sweet, flour-based dishes, such as pancakes or fried apple pancakes, into their diet during the pandemic. The increased consumption of sweet dishes during the pandemic may be associated with the stress of living in these unusual conditions. This finding is consistent with those of earlier studies, which showed that for many people, stress alters food selection in favor of a greater proportion of consumed calories coming from highly palatable foods (i.e., tasty, calorically dense foods containing high amounts of sugars, other carbohydrates and/or fats) [28].

A review article published by Clemente-Suárez, V.J. et al. showed results similar to those of our study. Those authors concluded that the COVID-19 lockdown promoted unhealthy dietary changes and increases in body weight of the population [29]. Contrary to our study, studies in Quebec adult residents found that diet quality had slightly improved during the COVID-19 early lockdown. There was a small improvement in the healthy eating index due to small increases in the consumption of recommended food products, such as whole grains, greens and beans, total vegetables and total dairy. The consumption of added sugar and refined grains had increased [30].

Evidence suggests that even a small positive energy balance over time is sufficient to cause weight gain in many individuals [31]. The mean increase in daily calorie intake in the BMI > 30 kg/m^2^ subgroup was approximately 320 kcal/d. This amount of additional calories accumulated over the course of a month corresponds to the weight gain of approximately 1.5 kg, which was observed and reported in an Italian study [4].

Dietary changes introduced by respondents with obesity during the pandemic have resulted not only in a significant increase in the calorie content of the diet but also a worsening of the NHDI. The PHNI showed no changes between the evaluated periods. These results are consistent with those observed in earlier studies, which demonstrated that the diets of respondents, particularly respondents with obesity, became worse during the pandemic [9]. People with obesity were shown not only to consume more food but also to have a greater tendency to consume meat, sweets, salty snacks and fast foods every day, while reducing the consumption of healthy products, such as fruit, vegetables and legumes. One Italian study also demonstrated that a higher BMI, as well as a younger age, were associated with an increase in junk food consumption (packaged sweets and baked products, sweet beverages, savory snacks and salad dressings) [32].

Despite the fact that respondents with BMI < 30 kg/m^2^ had started to pay more attention to the quality of their diet—as demonstrated by a reduced consumption of refined grains, lard, processed meat and red meat—these changes failed to improve their total NHDI scores. Similarly, the calorie intake in this patient population had not changed. This may be a reflection of the respondents’ efforts to maintain a steady body weight, which may have proved insufficient in preventing weight gain with the simultaneously reduced levels of physical activity. Nutritional changes during the COVID-19 pandemic have also been reported by other authors [33,34].

A comparison between the diets of individuals with BMI < 30 kg/m^2^ and BMI ≥ 30 kg/m^2^ may suggest that respondents with obesity began to pay less attention to good nutrition during the pandemic, unlike respondents without obesity who likely tried to reduce their food intake. This is supported by the observed change in the calorie content of their diets. Prior to the pandemic, individuals with BMI ≥ 30 kg/m^2^ consumed fewer calories than those with BMI < 30 kg/m^2^. During the pandemic, the situation became reversed, as respondents without obesity did not change their calorie intake, whereas those with BMI ≥ 30 kg/m^2^ started to consume more calories. This change is likely to have been even greater, since studies on nutritional habits show that the problem of underreporting portion sizes and omitting some products is more common among individuals with higher BMI values [35].

Although the purpose of our study was to assess the effects of obesity on changes in nutrition and physical activity, we also noticed the interesting phenomenon of decreased physical activity in people without obesity. Nonetheless, the pandemic seems not to have affected the level of physical activity in those with BMI > 30 kg/m^2^, with this group showing only a trend (*p* < 0.1) toward reduced physical activity during leisure time. Robinson et al. and Giustino et al. observed lower physical activity during lockdown in individuals with increased body weight [36,37]. Other authors emphasized lower levels of physical activity due to lockdown across all age groups, irrespective of BMI values [38,39]. Dunton et al. reported the lowest declared level of physical activity in the unemployed [40], which suggests a role of socioeconomic factors on the level of physical activity. Other factors that may have affected the level of physical activity during the pandemic include depression symptoms and lower mood [38]. Robinson et al. reported that respondents with BMI ≥ 35 kg/m^2^ declared a considerable drop in physical activity and identified three barriers that hindered maintaining a steady body weight: difficulties in the access to healthy food, lack of motivation and lack of social support [36]. However, our findings showed no significant changes in the level of physical activity declared by respondents with obesity during the pandemic, which may be explained by the usually low levels of physical activity in individuals with excessive body weight. Cross-sectional studies in the Polish adult population showed that 57.9% of respondents indicated moderate (0.5–2 h/day) and 16% of respondents indicated high (>2 h/day) levels of physical activity during the pandemic. Moreover, 43.3% of respondents reported that their level of physical activity became lower, and only 19.1% of respondents reported an increased level of physical activity in comparison with that before the pandemic [41]. Other studies in the Polish population showed that 34% of women reported weight gain during the pandemic and attributed it to unhealthy eating and lower levels of physical activity [42]. Despite the well-known potential inaccuracies typical of data collection methods reliant on self-reported levels of physical activity, questionnaire-based data collection has become one of the most common methods used in observational population studies in light of the limitations of lockdown settings.

A systematic review by Stockwell et al. demonstrated lowered levels of physical activity and an increased proportion of activities performed while seated in all ethnicities, age groups and irrespective of comorbidities during lockdown [43]. In one of our studies, all study groups, irrespective of the BMI value, showed a decrease in the proportion of individuals declaring moderate and high levels of physical activity during leisure time during lockdown, which may have resulted in a further increase in BMI values. Health clubs, or gyms, play an important role in increasing the level of physical activity [44]. Therefore, their closure in Poland over the entire period of the pandemic may have been associated with decreased levels of physical activity, particularly in those who had earlier declared a considerable proportion of their physical activity being moderate or high. However, our studies showed no changes in the declared time spent by respondents with obesity on sedentary activities during the pandemic.

To the best of our knowledge, previous studies indicating changes in the level of physical activity and food intake from the pre-pandemic period to the pandemic period were conducted during the pandemic, thus the pre-pandemic data were based on the respondents’ memories about their food consumption several weeks or months earlier [41,45]. Such nutritional studies based on retrospective methods involve an inherent bias due to the respondents’ imperfect recall of the facts on the frequency of food intake and portion sizes.

In general, recall bias is worse when respondents are asked to recall facts over longer periods. The longer the time period is, the more likely patients are to “telescope” forward or backward or to believe the event being recalled occurred more or less recently than it did [46]. Therefore, the further back the respondents must reach with their memories, the greater the risk of inaccurate data due to the respondents omitting some consumed products, meals, dishes or drinks. Moreover, subjects who do not have regular eating habits will have difficulty describing the “usual” frequency of consumption [47].

Due to the fact that there was an epidemiological study on food intake and physical activity levels scheduled for the years 2017–2020, we had a unique opportunity to compare the data obtained at two different points in time, i.e., a pre-pandemic period and a period during the pandemic, which had not been performed.

Although the respondents were not the same, the statistical tools used in the study helped select such respondents from the pre-pandemic period that had a comparable distribution of key factors that could affect eating behaviors and physical activity levels. The comparability of study groups was additionally verified by assessing the rates of selected medical conditions. All study subgroups showed the same rates of cardiovascular, respiratory, gastrointestinal, endocrine and musculoskeletal conditions, as well as malignancies, diabetes and allergy, except the BMI < 30 kg/m^2^ group in terms of respiratory and gastrointestinal conditions. Nonetheless, the rates of such conditions in those groups were low and ranged from 1% to 4%. This consistency in terms of the rates of medical conditions shows that the PrePandemic and Pandemic study groups were comparable.

Our study also had some limitations. One of them was the change in the methods of collecting nutritional interview data from direct (in person) to remote (via telephone). The telephone survey may be characterized by lower reliability of the collected data compared to the paper and pen interview. The interviewer has less control over the respondent’s understanding of the content of the questions. The interviewer cannot assess the level of understanding or hesitation in giving answers by the interview participant on the basis of non-verbal behavior. A health survey is not an ideal tool. The participant may respond according to the researcher’s expectations and not according to the actual state of affairs. During the pandemic period, the current anthropometric measurements were declared by respondents. However, the respondents were not asked about the exact measurement time. The rates of medical conditions were based on respondent declarations. The levels of physical activity were assessed with a questionnaire. Another limitation of the study was the fact that the diet indicators we used take into account only the most important groups of products. The healthy and unhealthy groups of products were selected on the basis of a literature review.

Moreover, we did not assess changes in respondent body weight. The rates of medical conditions were based on respondent declarations. The levels of physical activity were assessed with a questionnaire.

Considering the fact that obesity treatment is very difficult during the pandemic due to limited access to healthcare facilities, which are to a large extent occupied by COVID-19 patients [48], and limited access to medical treatment of obesity due to the lack of state-reimbursement for anti-obesity drugs, the only ways of halting obesity development are dietary and behavioral interventions. The nutritional changes we observed in individuals with BMI ≥ 30 kg/m^2^ clearly indicate the direction of the desired dietary and behavioral intervention. One additional finding is the possibility to limit the effects of the pandemic on individuals with BMI < 30 kg/m^2^, since, as our study demonstrated, the aim of intervention should be to restore the pre-pandemic levels of physical activity in this population.

Based on the results of our study, it can be concluded that the prevention and treatment of obesity in situations such as a pandemic or a stay-at-home order should be individualized depending on the presence or absence of obesity. In the case of people who do not suffer from obesity, our efforts should focus on maintaining physical activity prior to the pandemic (home isolation). In the case of people who suffer from obesity, efforts should be made to prevent increased calorie consumption.

## Figures and Tables

**Table 1:** Components of the total scores for pro-healthy and non-healthy diet.

Group of Products	Consumption Frequency (*)
Healthy groups of products—Pro-Healthy Diet (the higher the score, the better the nutrition)	
1. Vegetables	Several times a day
2. Fruits	At least once daily
3. Whole grains	At least once daily
4. Dairy products	At least once daily
5. Legumes	At least several times a week
6. Fish	At least once a week
7. Water	At least once daily
8. White meat	Several times a week or less frequently
Unhealthy groups of products—Non-Healthy Diet (the higher the score, the worse the nutrition)	
1. Refined grains	Several times a day
2. Red and processed meat	At least once a week
3. Canned meat	At least once a week
4. Butter	At least several times a week
5. Lard	At least several times a week
6. Fried food	At least once a week
7. Fast food	At least once a week
8. Sweets	At least several times a week
9. Salty snacks	At least several times a week
10. Sweetened drinks	At least once a week
11. Energy drinks	At least once a week
12. Alcohol	At least once a week

* The consumption of each product/food type with the indicated frequency yields 1 point.

**Table 2:** Physical activity assessment based on the KomPAN questionnaire [26].

Physical Activity Domain	The Level of Physical Activity		
	Low	Moderate	High
at work or in school	over 70% of time sitting	about 50% of time sitting and about 50% of time moving about	about 70% of time moving about or physical labor
during leisure time	mostly sedentary, watching TV, reading newspapers/books, light housework, walking for 1–2 h/week	walking, cycling, exercise, gardening or other light physical activity for 2–3 h/week	cycling, running, gardening and other sport/recreational activities that require physical activity for longer than 3 h/week

**Table 3:** Study group characteristics.

Variable *	BMI (kg/m^2^)	PrePandemic (*n* = 482)	Pandemic (*n* = 482)	χ^2^	*p*-Value **
Sex					
Male	<30	210 (83.00)	208 (82.21)	0.055	0.815
	≥30	43 (17.00)	45 (17.79)		
Female	<30	150 (65.50)	169 (73.80)	3.729	0.053
	≥30	79 (34.50)	60 (26.20)		
Education					
Primary/Junior high school	<30	23 (71.88)	21 (75.00)	0.075	0.785
	≥30	9 (28.13)	7 (25.00)		
Basic vocational	<30	128 (69.95)	147(78.19)	3.288	0.070
	≥30	55 (30.05)	41 (21.81)		
High school	<30	136 (73.91)	143 (79.01)	1.314	0.252
	≥30	48 (26.09)	38 (20.99)		
Higher	<30	73 (87.95)	66 (77.65)	3.122	0.077
	≥30	10 (12.05)	19 (22.35)		
Marital status					
Single	<30	70 (92.11)	69 (84.15)	2.362	0.124
	≥30	6 (7.89)	13 (15.85)		
Married\Civil partnership	<30	251 (74.70)	245 (78.53)	1.317	0.251
	≥30	85 (25.30)	67 (21.47)		
Divorced\Separated	<30	14 (63.64)	21 (80.77)	1.771	0.183
	≥30	8 (36.36)	5 (19.23)		
Widowed	<30	25 (52.08)	42 (67.74)	2.786	0.095
	≥30	23 (47.92)	20 (32.26)		
Household financial situation					
Good	<30	326 (74.43)	354 (79.91)	3.218	0.073
	≥30	112 (25.57)	89 (20.09)		
Bad	<30	34 (77.27)	23 (58.97)	3.757	0.053
	≥30	10 (22.73)	16 (41.03)		
Number of people in the household					
1	<30	53 (76.56)	64 (78.05)	0.256	0.613
	≥30	28 (23.44)	17 (21.95)		
>1	<30.00	307 (65.43)	313 (79.01)	3.723	0.054
	≥30.00	94 (34.57)	88 (20.99)		

* *n* (%); ** chi-squared test.

**Table 4:** Comparison of the rates of selected conditions between study groups.

Condition Types	BMI (kg/m^2^)	Affected	PrePandemic (*n* = 482)		Pandemic (*n* = 482)		χ^2^	*p*-Value *
			*n*	%	*n*	%		
Cardiovascular conditions	<30	No	304	84.44	316	83.82	0.054	0.817
		Yes	56	15.56	61	16.18		
	≥30	No	72	59.02	70	66.67	1.410	0.235
		Yes	50	40.98	35	33.33		
Respiratory conditions	<30	No	356	98.89	361	95.76	6.846	0.009
		Yes	4	1.11	16	4.24		
	≥30	No	114	93.44	102	97.14	1.676	0.196
		Yes	8	6.56	3	2.86		
Gastrointestinal conditions	<30	No	356	98.89	364	96.55	4.464	0.035
		Yes	4	1.11	13	3.45		
	≥30	No	113	92.62	101	96.19	1.330	0.249
		Yes	9	7.38	4	3.81		
Endocrine conditions (excluding diabetes)	<30	No	353	98.06	363	96.29	2.082	0.149
		Yes	7	1.94	14	3.71		
	≥30	No	107	87.70	98	93.33	2.043	0.153
		Yes	15	12.30	7	6.67		
Musculoskeletal conditions	<30	No	328	91.11	339	89.92	0.304	0.582
		Yes	32	8.89	38	10.08		
	≥30	No	110	90.16	90	85.71	1.066	0.302
		Yes	12	9.84	15	14.29		
Malignancies	<30	No	358	99.44	374	99.20	0.158	0.691
		Yes	2	0.56	3	0.80		
	≥30	No	120	98.36	103	98.10	0.023	0.880
		Yes	2	1.64	2	1.90		
Diabetes	<30	No	345	95.84	363	96.30	0.100	0.752
		Yes	15	4.16	14	3.70		
	≥30	No	105	86.10	91	86.70	0.017	0.895
		Yes	17	13.90	14	13.30		
Allergy	<30	No	347	96.39	360	95.49	0.380	0.537
		Yes	13	3.61	17	4.51		
	≥30	No	116	95.08	102	97.14	0.629	0.428
		Yes	6	4.92	3	2.86		

* chi-squared test.

**Table 5:** Assessment of physical activity during leisure time.

BMI (kg/m^2^)	Level of Physical Activity	PrePandemic (*n* = 482)			Pandemic (*n* = 482)			(z) *p*-Value *
		*n*	%	(z) *p*-Value *	*n*	%	(z) *p*-Value *	
<30	Low	105	29.2	(4.693) <0.001	179	47.5	(3.145) 0.002	(5.969) <0.001
	Moderate	209	58.1		185	49.1		
	High	46	12.8		13	3.4		
>30	Low	65	53.3		67	63.8		(1.937) 0.053
	Moderate	49	40.2		38	36.2		
	High	8	6.6		0	0.0		

* Mann–Whitney–Wilcoxon test; Low: mostly sedentary, watching TV, reading newspapers/books, light housework, walking for 1–2 h/week; Moderate: walking, cycling, exercise, gardening or other light physical activity for 2–3 h/week; High: cycling, running, gardening and other sport/recreational activities that require physical activity for longer than 3 h/week.

**Table 6:** Assessment of physical activity at work or school.

BMI (kg/m^2^)	Level of Physical Activity	PrePandemic (*n* = 233#)			Pandemic (*n* = 261#)			(z) *p*-Value *
		*n*	%	(z) *p*-Value *	*n*	%	(z) *p*-Value *	
<30	Low	46	23.5	1.840 (0.066)	62	29.0	(2.300) 0.021	(2.001) 0.045
	Moderate	101	51.5		116	54.2		
	High	49	25.0		36	16.8		
>30	Low	14	37.8		23	48.9		(0.974) 0.330
	Moderate	17	45.9		18	38.3		
	High	6	16.2		6	12.8		

* Mann–Whitney–Wilcoxon test; # The level of physical activity at work/school was assessed only in respondents who were working or studying at the time; Low: mostly sedentary, watching TV, reading newspapers/books, light housework, walking for 1–2 h/week; Moderate: walking, cycling, exercise, gardening or other light physical activity for 2–3 h/week; High: cycling, running, gardening and other sport/recreational activities that require physical activity for longer than 3 h/week.

**Table 7:** Assessment of the effects of obesity on the quality of nutrition in the pre-pandemic and pandemic periods.

	BMI	PrePandemic (*n* = 482)			Pandemic (*n* = 482)			*p*-Value **
		M	SD	*p*-Value *	M	SD	*p*-Value *	
Pro-Healthy Diet Indicator	<30	2.92	1.43	0.294	2.73	1.30	0.970	0.057
	≥30	2.77	1.41		2.72	1.18		0.796
Non-Healthy Diet Indicator	<30	4.84	2.13	0.003	4.67	2.01	0.685	0.267
	≥30	4.06	1.48		4.58	2.16		0.049

M—mean, SD—standard deviation; * post hoc LSD test for comparison <30 vs. ≥30 sample; ** post hoc LSD test for comparison of PrePandemic vs. Pandemic samples.

**Table 8:** Changes in the consumption frequency of selected foods from the Non-Healthy Nutrition Indicator during the pandemic.

Food Types	BMI	PrePandemic (*n* = 482)		Pandemic (*n* = 482)		*p*-Value
		M	SD	M	SD	
Refined grains	<30	1.37	0.77	1.20	0.79	0.003
	≥30	1.27	0.78	1.34	0.76	0.49
Fried foods	<30	0.35	0.23	0.35	0.24	0.74
	≥30	0.30	0.20	0.38	0.33	0.002
Lard	<30	0.20	0.29	0.12	0.24	0.000
	≥30	0.10	0.17	0.11	0.18	0.83
Processed meat	<30	0.95	0.69	0.74	0.54	0.000
	≥30	0.78	0.54	0.86	0.56	0.32
Red meat	<30	0.32	0.24	0.26	0.22	0.000
	≥30	0.28	0.21	0.28	0.24	0.8

**Table 9:** Daily calorie (kcal/day) and macronutrient (g/day) intake in respondents’ diets.

	BMI (kg/m^2^)	PrePandemic		Pandemic		*p* **
		M ± SD	*p* *	M ± SD	*p* *	
Calories (kcal)	<30	2194 ± 789	0.005	2217 ± 695	0.370	0.680
	≥30	1971 ± 880		2290 ± 591		0.001
Total carbohydrates (g)	<30	283.1 ± 96.8	0.002	280.2 ± 93.3	0.060	0.670
	≥30	252.0 ± 101.5		299.3 ± 83.8		0.000
Sucrose (g)	<30	58.3 ± 33.0	0.009	62.6 ± 39.5	0.030	0.120
	≥30	51.7 ± 30.0		71.6 ± 49.9		0.000
Plant protein (g)	<30	29.8 ± 11.4	0.002	29.00 ± 10.8	0.820	0.290
	≥30	26.3 ± 12.1		29.3 ± 8.3		0.040
Total fat (g)	<30	80.2 ± 36.6	0.053	82.6 ± 30.9	0.557	0.335
	≥30	73.1 ± 42.6		84.9 ± 29.6		0.011
Saturated fatty acids (g)	<30	30.8 ± 14.5	0.421	32.4 ± 13.3	0.247	0.112
	≥30	29.5 ± 16.4		34.3 ± 13.9		0.014
Total protein (g)	<30	82.7 ± 31.8	0.055	82.6 ± 25.1	0.976	0.993
	≥30	76.8 ± 37.8		82.7 ± 22.3		0.128
Animal protein (g)	<30	51.7 ± 23.6	0.510	52.4 ± 9.5	0.999	0.637
	≥30	50.1 ± 29.2		52.4 ± 17.7		0.434
Dietary fiber (g)	<30	18.5 ± 6.5	0.176	17.9 ± 6.5	0.395	0.218
	≥30	17.6 ± 7.6		18.5 ± 5.4		0.276

M—mean, SD—standard deviation; * post hoc LSD test for comparison <30 vs. ≥30 sample; ** post hoc LSD test for comparison of PrePandemic vs. Pandemic samples.

## Data Availability

The data presented in the analysis are the property of the Polish Ministry of Health. The authors of the article, as the researchers responsible for the study, used them on the basis of the separate agreement with Ministry of Health.
